# On a New Amalgam for the Teeth

**Published:** 1849-07

**Authors:** 


From the Dental Recorder.

### ON A NEW AMALGAM FOR THE TEETH.

The following articles are taken from the London Lancet, and that our readers may have every thing new connected with the practice of Dental Surgery, we publish them, "the bane and antidote," before testing the properties of the new mixture for filling teeth. It will be seen that the amalgam controversy is not confined to our own country, and that the arguments made use of in England against it are about the same as in America. No new light is thrown upon the subject by Mr. Levison, who is in such a hurry to condemn the new amalgam, that he cannot wait to test its qualities. It strikes us that it will be well for those who are opposed to the use of amalgams, to try this new one before condemning it, for if, as Mr. Evans says, "it retains its color perfectly, neither oxydizing on the external surface, nor on that applied to the cavity;" the objection that it may produce salivation would appear to have but little force, as it is generally contended that ptyalism can only be produced by amalgam fillings, in consequence of the oxyd which is formed being taken up by the absorbents.—*N. Y. Dental Recorder.*

"*To the Editor of the Lancet:*

"Sir:—I shall be obliged if you will allow me, through the medium of your journal, to make known to my professional brethren the composition of an amalgam invented by myself some years ago, which I have used with much success for a length of time, in some peculiar cases, and have experimented with it extensively in filling carious teeth. It is composed of chemically pure tin, prepared with much care, to insure its being free from any other metalic substance, and combined with prepared cadmium, in small quantities, and mercury. In using it, more or less mercury should be employed, as may be required to make it more or less plastic.

"The cavity of the tooth being previously thoroughly freed from carious matter, can be carefully filled with the paste thus formed. In the course of a few minutes it hardens into a solid, and gradually acquires a still firmer consistency and toughness, exhibiting a whitish color, or, if cut or burnished, a metallic lustre, like that of pure tin.

"The advantages of this filling, I believe, are such as are possessed by no other amalgam. It retains its color perfectly, neither oxydizing on the external surface, nor on that applied to the cavity, and of course it does not discolor the tooth itself. It fills each crevice of the cavity, and, effectually excluding moisture, and all kinds of deleterious matters, prevents the recurrence of caries; and becomes sufficiently hard to withstand the friction of mastication. To these most important advantages may be added others—*e. g.*, it is easily and quickly prepared, without the trouble of heating it, as is the case with some of the amalgams hitherto used. It is readily applied to the cavity of the teeth, and without the disagreeable creaking sound which attends the employment of other preparations. It will not amalgamate with, or injure any gold claps or plate bearing artificial teeth, which may be placed in contact with it; and in case of its removal being necessary, it can be cut out as easily as a good filling, as it forms a tough, almost ductile substance, and not a hard, brittle one, like the ordinary amalgams.

"I have submitted it to the inspection and trial of some of the best dentists here and in London. As far as their opportunities of investigating it have hitherto extended, I think they fully agree with me as to its advantages. It is, I believe, the best filling hitherto used, in those cases where amalgams are thought to be useful; and some of my friends are willing to award it even higher praise than this.

"Believing it, therefore, to be a useful discovery, I wish to place it in the hands of the profession, and I make this communication to you, at the same time that I publish a similar one in France, and in my native country, America, where I first used it.

I am, Sir, your obedient servant,

Thomas W. Evans, Dentist."

